# CAN VIRTUAL REALITY BE AS GOOD AS OPERATING ROOM TRAINING? EXPERIENCE FROM A RESIDENCY PROGRAM IN GENERAL SURGERY

**DOI:** 10.1590/0102-672020180001e1397

**Published:** 2018-12-06

**Authors:** Bruno Della Mea GASPERIN, Thamyres ZANIRATI, Leandro Totti Cavazzola

**Affiliations:** 1 Department of Surgery, Hospital de Clínicas de Porto Alegre, Universidade Federal do Rio Grande do Sul, Porto Alegre, RS, Brazil

**Keywords:** Virtual reality, General surgery, Medical education, Treinamento por simulação, Cirurgia geral, Educação Médica

## Abstract

**Background:**

The increasingly intense usage of technology applied to videosurgery and the advent of robotic platforms accelerated the use of virtual models in training surgical skills.

**Aim:**

To evaluate the performance of a general surgery department’s residents in a video-simulated laparoscopic cholecystectomy in order to understand whether training with virtual reality is sufficient to provide the skills that are normally acquired in hands-on experience at the operating room.

**Methods:**

An observational study with twenty-five first- and second-year general surgery residents. Each subject performed three video-laparoscopic cholecystectomies under supervision in a simulator. Only the best performance was evaluated in the study. Total number of complications and total procedure time were evaluated independently. The groups were defined according to total practice time (G1 and G2) and the year of residency (R1 and R2), each being analysed separately.

**Results:**

Twenty-one residents finished the three practices, with four follow-up losses. Mean practice time was 33.5 hours. Lowering of the rate of lesions in important structures could be identified after a level of proficiency of 60%, which all participants obtained regardless of previous in vivo experience. No significant difference between the R1 and R2 groups was observed.

**Conclusion:**

Learning in groups R1 and R2 was equal, regardless of whether previous practice was predominantly in vivo (R2) or with virtual reality (R1). Therefore, it is possible to consider that skills obtained in virtual reality training are capable of equalising the proficiency of first- and second-year residents, being invaluable to increase patient safety and homogenise learning of basic surgical procedures.

## RESUMO

**Racional:**

O uso cada vez mais intenso da tecnologia aplicado à cirurgia em vídeo e o advento das plataformas robóticas, aceleraram o uso de modelos virtuais no treinamento de habilidades cirúrgicas.

**Objetivo:**

Avaliar o desempenho dos médicos residentes em um serviço de cirurgia geral em colecistectomia vídeo simulada laparoscópica em um centro de realidade virtual para entender se o treinamento de realidade virtual é suficiente para equipará-lo às habilidades adquiridas no centro cirúrgico.

**Método:**

Estudo observacional transversal com 25 residentes de cirurgia geral do primeiro e segundo anos. Cada residente realizou três colecistectomias videolaparoscópicas com supervisão em um simulador. O melhor desempenho foi avaliado no estudo. O número total de complicações e tempo total do procedimento foram avaliados de forma independente. Os grupos foram definidos de acordo com o tempo total de prática (G1 e G2) e o ano de residência (R1 e R2), os quais foram analisados isoladamente.

**Resultados:**

Vinte e um médicos residentes médicos concluíram as 3 práticas, com 4 perdas de seguimento e praticaram uma média de 33,5 h. Diminuição das lesões em estruturas importantes foi identificada após nível de proficiência de 60%, que todos os participantes obtiveram independentemente da experiência anterior in vivo. Não houve diferença significativa entre os resultados dos grupos R1 e R2.

**Conclusões:**

O aprendizado dos grupos R1 e R2 pode ser considerado igual, independentemente de a prática anterior ser majoritariamente in vivo (R2) ou em realidade virtual (R1). Assim, é possível considerar que as habilidades cirúrgicas adquiridas a partir do treinamento virtual são capazes de equiparar a proficiência dos residentes de primeiro e segundo ano, sendo fundamental para aumentar a segurança dos pacientes e homogeneizar o aprendizado de procedimentos cirúrgicos básicos.

## INTRODUCTION

Traditionally, medical residents of surgical specialties were taught in the classical way based on the teachings of Halsted, summarized as “see once, perform once, and teach once”^10^, under the supervision of a trained surgeon. Nevertheless, using the “Halsted training method” in a non-specialized centre may take each resident a different amount of time to consolidate the knowledge needed for each procedure in a heterogeneously effective way^9^. It is believed that around 30 laparoscopic cholecystectomies are necessary for a surgeon to be considered able of performing such a procedure safely, for the risk of damaging vital structures, especially the cystic duct, that falls drastically after this number^13^; thus the Halstedian model is not enough for the patient safety. Furthermore, the ethical implications of learning using humans and the legal risks during such a process are to be considered. Training, particularly in laparoscopic surgery, must be done in a step-wise fashion of increasing difficulty, first encompassing skills that are fundamental to understand the tools used during the procedure, then the procedure in itself, according to the level of complexity^9^ ^,^ ^14^. In this way, virtual reality (VR) is an educational tool of great potential, able to provide practice in an ambient controlled and free from the pressure of the operating room^1^, and, as shown in a systematic review of 22 studies with 622 participants, to enhance the training of basic skills that will be transferred to the operating room practice^19^. 

The aim of this study was to investigate whether VR training could equalize the results of first- and second-year general surgery residents on laparoscopic cholecystectomy, comparing the results obtained in performing it in a VR simulator both by first-year (who have performed it almost exclusively in the simulator) and second-year residents (whose major part of training took place in the operating room). 

## METHODS

This study was approved by the Hospital de Clínicas de Porto Alegre Ethics Committee under the number #5327. 

**FIGURA 1:**
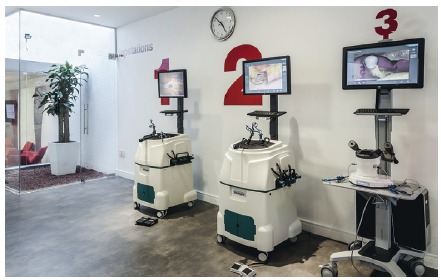
Simulator (haptic model, Simbionix Inc, corner of Golan and Hagenev streets, Airport City, Israel)

It is a cross-sectional observational study of twenty-five first- and second-year general surgery residents in a university hospital in Porto Alegre, performed between March and December 2015. During these months, all the residents could train their laparoscopic skills in an on-demand basis at a virtual reality centre bound to their home institution. They also received standard surgical training in the operating room. In December 2015, each of these residents was invited to perform three laparoscopic cholecystectomies in accordance with a standardized protocol and under supervision in a Mentor LAP simulator (haptic model, Simbionix Inc, corner of Golan and Hagenev streets, Airport City, Israel), as shown in this image (Figure 1).

The three cholecystectomies were performed in tandem, and only the best performance was analysed in the study. The best performance was defined according to ten pre-established parameters of proficiency taken together: non-cauterized bleeding, severe complications with potential damage to vital structures (e.g., cutting or cauterizing a duct or an artery before clipping; placing clips on the common bile duct or on the hepatic artery), cauterization efficacy, number of lost clips, safe usage of electrocautery, time of cauterizing without appropriate contact with adherences, time of cauterization performed at less than 15 mm from a clip, time of cauterization performed at less than 5 mm from the duct, total procedure time, and number of used clips. Each of those parameters amounted to 10% of the final score, which varied from 0 to 100 defining the proficiency at the single surgery. The first three parameters were defined, in the order presented above, as tie-up criteria to establish a better and a worse try when two would present the same proficiency score. Total number of complications and total procedure time were assessed independently. 

The participants were divided in two groups according to the level of virtual reality training from March 2015 through December 2015 (G1 and G2), and further in two other groups according to which year in the residency program they were at the time (R1 or R2)

**FIGURE 2:**
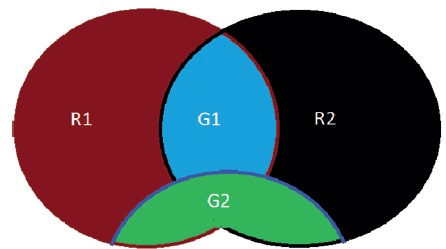
Division of groups

The ideal total training time suggested to the participants in the beginning of the study was 72 h (2 h weekly for nine months). The first group (G1) included those that practice 50% (36 h) or less of the recommended time; residents with more than 36 h of training were allocated to the second group (G2). The relationships between the procedure performance and the other variables were analyzed by observing the number of hours spent on virtual reality training (G1 vs. G2) and the year of postgraduate training (R1 vs. R2, Figure 2). 

R1 - first year residents; R2 - second year residents; G1 - residents with training time less than 50% of the ideal; G2 - residents with training time greater than 50% of the ideal.

### Statistical Analysis

The chi-square and the Mann-Whitney U-test were performed on SPSS (IBM Inc). For data with non-parametric distribution, median and interquartile range were used.

## RESULTS

No significant statistical difference in proficiency level obtained by participants of either R1 or R2 was observed. Median proficiency was 60% (40-100%), as measured by the simulator. It is of notice that the chosen procedure - laparoscopic cholecystectomy - must be performed, as determined by the National Committee on Medical Residency, in the second year of residency^16^; this allows us to presume that, in general, the major experience of the R1 group with it was in virtual reality, whilst it was the operating room for the R2 group. In our centre, first-year residents that display the necessary skills before the end of the year may be allowed to perform this procedure. Reviewing the data regarding performed surgeries during the study period in our centre, we found that the participants in the R1 group performed 5-9 laparoscopic cholecystectomies, whilst the ones in the R2 group performed around 100. 

No significant statistical difference in total procedure time was found between the participants. Furthermore, after a proficiency of 60%, regardless of the main method of training (virtual reality or operating room), there was a significant reduction (p=0.001) of damage to the vital structures, which occurred in 19% of the cases. At the end of the study period, we noticed that the actual mean time of training (33.5 h) was lower than the ideal one; however, there was not sufficient statistical power, due to the sample size, to detect a significant difference between G1 and G2. The residents (n=2) that obtained the best proficiency scores (90% and 100%), practiced, respectively, 92 and 88 h in the simulator, according to its registry. We had four follow-up losses, which were excluded from the results. 

## DISCUSSION

Simulation is being increasingly recognised as a valid way of learning. It has a growing importance for the safe and effective development of surgical skills, bringing better results than traditional methods of teaching^12^. It offers a lower-pressure environment, lowers costs, and solves ethics dilemmas related to the use of animal and human models^9^. 

The aims of deliberate simulation practices, such as virtual reality, are to promote constant improvement and keeping of abilities. In this way, it is of utmost importance for the training to be continuously performed for at least 2 h a week, as recommended by the Royal College of Surgeons^6^. Nevertheless, despite the recommended time of practice being so, the participants did not achieve it, regardless of constant encouraging. This result may have been influenced by the way of teaching and learning in the Medicine undergraduate period - which is almost entirely based on in vivo practice - that may render some people sceptical^3^ of the transmission of skills by simulated operating rooms, in spite of known evidences^2^ ^,^ ^4^ ^,^ ^5^ ^,^ ^8^ ^,^ ^15^ ^,^ ^18^ ^,^ ^20^. Another relevant fact is that this training was not mandatory in the residency program - this was changed after the conclusion of this study, as the importance of virtual reality practice was clearly demonstrated. Furthermore, the format of feedback may be relevant^3^, since virtual reality information is released at the end of the procedure, whilst it occurs real-time during actual operations. 

In the university hospital where this study was performed there is a simulation centre that provided the participants with a pressure-free, safe, well-lit, air-conditioned, and easy-accessible environment. In it, different skill levels may be learned in a way that failure does not mean harm, which allows the students to concentrate all their attention into the ongoing task and so optimize their training time. Besides, a tutor reviews their performance and makes weekly and monthly commentaries. This results allow us to infer that first-year resident physicians reached the same proficiency level as the second-year ones due to the significantly greater time of virtual reality training, regardless of previous practise (i.e., in vivo, with virtual realities, or with videogames^11^) - these data were not analyzed in this study. Thus, it is also possible to infer that virtual reality practice seems to even the skills of participants in both the R1 and R2 groups of performing a simulated laparoscopic cholecystectomy. This could mean a better prepare for performing in vivo procedures in the R1 group and, as consequence, greater confidence for the surgeon and safety for the patients. This must be determined, however, by further, more specific studies that are beyond the scope of this article. Lastly, virtual reality could be useful to increase the students’ exposure to surgeries not so commonly performed, helping them obtain the necessary proficiency to overcome the learning curve and reducing the previously described after-graduation insecurities^7^. Citing Dimitrios *et al*.^17^, it seems that the secular Halstedian training method should be reformulated to “see once, simulate thoughtfully, perform once”. 

In this way, we consider essential that a standardized virtual reality training plan be part of the residency programs of all surgical specialties, even if further studies are necessary to determine the minimal training time to achieve basic procedure proficiency in virtual reality. Bigger sample sizes are needed to evaluate precisely the impact of virtual reality on medical training and to confirm the relationship between practice time and proficiency in simulated laparoscopic cholecystectomy. 

## CONCLUSION

Learning in the R1 and R2 groups may be considered the same, regardless of previous practice being predominantly in viv*o* (R2) or in virtual reality (R1). Thus, it is possible to consider that surgical skills acquired through virtual training are capable of levelling the first- and second-year residents’ proficiency, being fundamental to increase patient safety and to homogenize learning of the basic surgical procedures. 
